# Effect of *N*-Glycan Profiles on Binding Affinity of Diagnostic Antibody Produced by Hybridomas in Serum-Free Suspension

**DOI:** 10.4014/jmb.2501.01036

**Published:** 2025-05-15

**Authors:** Tae-Ho Kim, Dae Eung Kim, Hoon-Min Lee, Mi-Jung Kang, Jung Hwa Kim, Jungmok You, Mi Kyeong Lee, Yeon-Gu Kim

**Affiliations:** 1 Biotherapeutics Translational Research Center, Korea Research Institute of Bioscience and Biotechnology (KRIBB), Daejeon 34141, Republic of Korea; 2 Department of Convergent Biotechnology & Advanced Materials Science, Graduate School of Biotechnology, College of Life Science, Kyung Hee University, Yongin-si 17104, Republic of Korea; 3 College of Pharmacy, Chungbuk National University, Cheongju 28160, Republic of Korea; 4 Department of Bioprocess Engineering, KRIBB School of Biotechnology, University of Science and Technology (UST), Daejeon 34113, Republic of Korea; 5 Department of R&D, Boditech Med Inc., Chuncheon 24398, Republic of Korea

**Keywords:** Hybridomas, diagnostic antibody, suspension culture, glycosylation, binding affinity

## Abstract

Serum-free suspension culture for hybridomas is one of the important key steps for efficient diagnostic antibody production while maintaining protein quality and function. Based on the importance of *N*-glycan profiles in therapeutic antibody production in mammalian cells, the effect of changes in the *N*-glycan profiles on the function of diagnostic antibody must also be validated. To investigate the influence of diagnostic antibodies with different *N*-glycan profiles on the binding affinity with target antigens, four glycosylation regulators, tunicamycin, Bis-Tris, galactose, and *N*-acetylmannosamine, were administered separately to diagnostic antibody-producing hybridomas cultures. Supplementation with these four glycosylation modulators inhibited glycosylation and increased mannosylation, galactosylation, and sialylation in serum-free suspended hybridomas. In particular, the diagnostic antibody produced from a culture with tunicamycin exhibited a significant increase in the aglycosylated form compared with those without tunicamycin or with other glycosylation modulators. Surprisingly, diagnostic antibody with different *N*-glycan compositions did not significantly affect binding affinity with the target antigen and even aglycosylated antibodies did not affect binding affinity. Taken together, the results indicate that the change in the *N*-glycan profile of the diagnostic antibody produced in serum-free suspension hybridomas in an altered culture environment did not significantly affect their biological function, which provides valuable insight for the production and quality control of diagnostic antibody.

## Introduction

Hybridomas are cells created by the fusion of monoclonal antibody-producing B cells and immortal myeloma cells [1]. The combined properties enable the hybridoma to consistently and reliably produce a specific monoclonal antibody derived from B cells. Because of this characteristic of hybridomas, which can produce an unlimited number of identical monoclonal antibodies, they are primarily used in diagnostic areas that require high specificity for specific target antigens [2]. One of the main challenges during the production of diagnostic antibody by hybridomas is the suspension culture using serum-free media, which has many advantages over the mouse ascites method-based production and serum-containing adherent-based production [3, 4]. The use of serum-free media prevents contamination with nonspecific monoclonal antibodies derived from mouse ascites and fetal bovine serum [4]. Adherent cultures are limited by the surface area available for cell attachment; however, suspension cultures of hybridomas are easier to scale up and achieve higher cell concentrations, which effectively increases volumetric antibody production [5]. Therefore, adapting hybridomas to serum-free suspension culture is a prerequisite for the efficient production of diagnostic antibody while maintaining protein quality.

For canonical antibody production using mammalian cells, the *N*-glycosylation pattern within the Fc region of the canonical antibody structure is significantly affected by the culture environment [6, 7]. For example, in recombinant Chinese hamster ovary (rCHO) cell cultures for the production of therapeutic proteins, including canonical antibody, impaired glycosylation can occur under various stress culture conditions [8, 9]. Stressful culture conditions can induce cell death by membrane disruption, resulting in the release of enzymes, such as proteases and glycosidases, which disrupt the glycosylation of extracellularly produced glycoproteins [10]. Because *N*-glycosylation for the biological function of therapeutic glycoproteins is important, the cell culture process for rCHO cells must be optimized, including feeding strategies for maintaining high cell viability to mitigate cell death and prevent *N*-glycan damage [11-13]. Supplementing rCHO cell culture with various nucleotide sugar precursors has been done to improve the *N*-glycosylation of therapeutic glycoproteins [14-17]. In contrast to efforts to modulate *N*-glycans in therapeutic canonical antibody production from mammalian cells, there is little evidence that the *N*-glycan profile of diagnostic antibodies produced in hybridomas is responsible for their function, particularly their binding affinity with the target antigen.

In this study, we examined the effect of diagnostic antibodies with different *N*-glycan profiles on their binding affinity with the target antigen. Diagnostic monoclonal antibodies with diverse *N*-glycan profiles were produced in serum-free suspension cultures of hybridomas supplemented with various glycosylation modulators. We analyzed *N*-glycan formation and intracellular nucleotide sugar levels by hydrophilic interaction high-performance liquid chromatography (HPLC) and ion-pair reversed-phase HPLC.

## Materials and Methods

### Cell line, Culture Maintenance, and Glycosylation Modulator Treatment

The diagnostic antibody-producing hybridomas, provided by Med Inc. (Republic of Korea), were initially maintained in an adherent culture using Dulbecco's modified Eagle's medium (Hyclone, USA) supplemented with 10% (v/v) fetal bovine serum (FBS; Thermo Fisher Scientific, USA). The hybridomas were then grown in a serum-free suspension culture using a Climo-shaking CO_2_ incubator (Adolf Kühner, Switzerland) at 110 rpm, 70% humidity, and 37°C. SFM4CHO (Hyclone) supplemented with 4 mM glutamine was used for culture maintenance.

Exponentially growing cells were seeded at a concentration of 3 × 10^5^ cells/ml in 125 ml Erlenmeyer flasks (Corning, USA) containing 30 ml of SFM4CHO supplemented with 4 mM glutamine and Cell Boost 6 (Hyclone). After 2 days, 1 μg/ml tunicamycin (Sigma-Aldrich, USA), 10 mM Bis-Tris (Sigma-Aldrich), 20 mM galactose (Sigma-Aldrich), and 20 mM *N*-acetylmannosamine (ManNAc; Sigma-Aldrich) were added individually to the culture as glycosylation modulators.

### Cell Concentration, Viability, Antibody Assay, and Amino Acid Analysis

The cell concentration and viability were evaluated using a Cedex HiRes analyzer (Roche Diagnostics, Switzerland) based on the trypan blue dye exclusion method. The concentration of the secreted diagnostic antibody in the culture supernatant was quantitated using a Cedex Bio analyzer (Roche Diagnostics) based on the manufacturer’s instructions. Amino acid concentrations in the spent media were measured using a REBEL cell culture analyzer (908 Devices, USA), a microfluidic capillary electrophoresis combined with high-pressure mass spectrometry. The samples were analyzed in technical triplicates with calibration curves automatically generated from triplicate analysis of the standards.

### *N*-Glycan Analysis of the Diagnostic Antibody

The diagnostic antibody was purified from the cell culture supernatants by protein G chromatography using a HiTrap Protein G HP (Cytiva, UK) and a liquid chromatography system (ÄKTA pure 25 L; Cytiva). The *N*-glycans of the purified diagnostic antibody were enzymatically released using PNGase F (Roche Diagnostics) and fluorescently labeled with 2-aminobenzamide (Sigma-Aldrich). The labeled *N*-glycans were injected directly into an AdvanceBio Glycan Map column (2.7 μm, 4.6 mm × 150 mm; Agilent Technologies, USA) connected to an Ultra HPLC (UHPLC) system (1290 Infinity II Bio LC system; Agilent Technologies). The labeled *N*-glycans were monitored using a fluorescence detector at an excitation wavelength of 260 nm and an emission wavelength of 430 nm.

### Quantitation of the Intracellular Nucleotide Sugars

Intracellular nucleotide sugars were analyzed as previously described [18]. Briefly, ion-pair reversed-phase chromatography was used for quantitating intracellular nucleotide sugars, which were detected at 254 nm using a Waters ACQUITY Premier HSS T3 column with VanGuard FIT (150 mm; Waters, USA) connected to a UHPLC system (1290 Infinity II Bio LC system; Agilent Technologies). Peak identification of the nucleotide sugars was achieved using a standard mixture containing the following nucleotide sugars: CMP-sialic acid (CMP-SA), UDP-galactose (UDP-Gal), UDP-glucose (UDP-Glc), UDP-*N*-acetylglucosamine (UDP-GlcNAc), and GDP-fucose (GDP-Fuc). The integrated peak areas of the standard mixture were calculated using the OpenLab CDS ChemStation software (Agilent Technologies) following UHPLC analysis.

### ELISA for Diagnostic Antibody with Specific Target Antigen

The purified diagnostic antibody was tested with the target antigen provided by Boditech Med Inc., (Republic of Korea) using a conventional ELISA, as described previously [19]. Briefly, 96-well plates (Corning) were coated with antigen in carbonate-bicarbonate buffer and incubated overnight at 4°C. After five washes with phosphate buffered saline (PBS; Sigma-Aldrich) containing 0.1% Tween 20 (Sigma-Aldrich), the plates were blocked with PBS containing 1% bovine serum albumin (Sigma-Aldrich). Synthetic buffer (WellChampion; Kem-En-Tec, Denmark) was then added to stabilize the wells, followed by incubation for 1 h at 37°C. Various concentrations of the purified diagnostic antibody and positive quality control materials, provided by Boditech Med Inc., were added to the wells and incubated for 1 h at 37°C. After washing, horseradish peroxidase (HRP)-conjugated anti-mouse IgG secondary antibody (KPL, USA) in HRP stabilizer (Kem-En-Tec) was added and incubated for 1 h at 37°C. The peroxidase reaction was visualized by adding TMB ONE (Kem-En-Tec) for 15 min at 25°C. Color development was stopped with the addition of H_2_SO_4_. The absorbance was measured at 450 nm.

### Statistical Analysis

The results are expressed as mean ± standard deviation. The data were analyzed using a two-tailed student’s *t*-test when appropriate. Differences between means were considered significant at *p* ≤ 0.05.

## Results and Discussion

### Effects of Glycosylation Modulators on Cell Growth, Antibody Production, and Metabolism

To determine the effect of glycosylation modulators on cell growth and antibody production in hybridomas, a diagnostic antibody-producing hybridomas was adapted to serum-free suspension culture and cultivated with glycosylation modulators. We selected four glycosylation modulators, tunicamycin, Bis-Tris, galactose, and ManNAc, which are known to inhibit glycosylation or increase mannosylation, galactosylation, and sialylation in mammalian cells, respectively [14, 19-21]. Cells without glycosylation modulators were also cultured as a control.

**Fig. 1:**
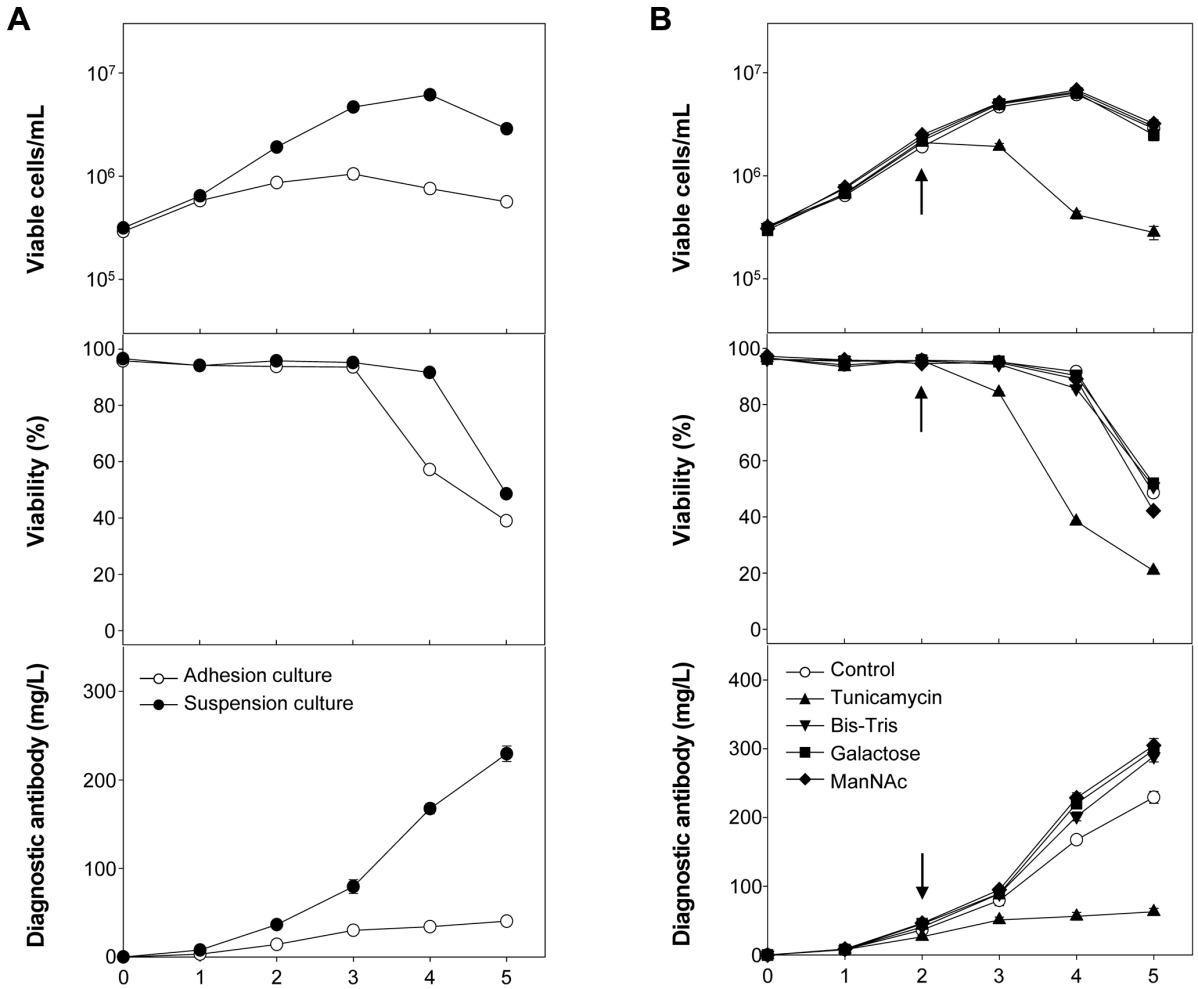
Profiles of cell growth, viability, and diagnostic antibody production from hybridomas (A) in serumcontaining adherent and serum-free suspended culture mode (B) with and without four glycosylation modulators. The arrow indicates the time of glycosylation modulator addition to the culture media. Error bars represent the standard deviations calculated from technical duplicate experiments.

Fig. 1 shows the profiles of viable cell concentration, viability, and diagnostic antibody concentration of serum-free suspended hybridomas during the culture with or without glycosylation modulators. From the serum-containing adherent culture to the serum-free suspension culture mode for diagnostic antibody-producing hybridomas, the maximum viable cell concentration and final antibody production increased by 4.5- and 5.7-fold, respectively (Fig. 1A). As shown in Fig. 1B, the culture treated with tunicamycin exhibited a marked decrease in cell viability and diagnostic antibody production. The same amount of solvent (DMSO) used to prepare the tunicamycin solution did not show any detrimental effects (*p* > 0.05) (data not shown). The remaining three glycosylation modulators increased diagnostic antibody production without improving cell growth, indicating that these treatments are effective at enhancing specific protein productivity (*q*_p_). Although the extent of the effectiveness varied depending on optimized culture conditions, the trends were similar to those of previous studies of the glycosylation modulators used in this study [14, 19-21].

**Fig. 2:**
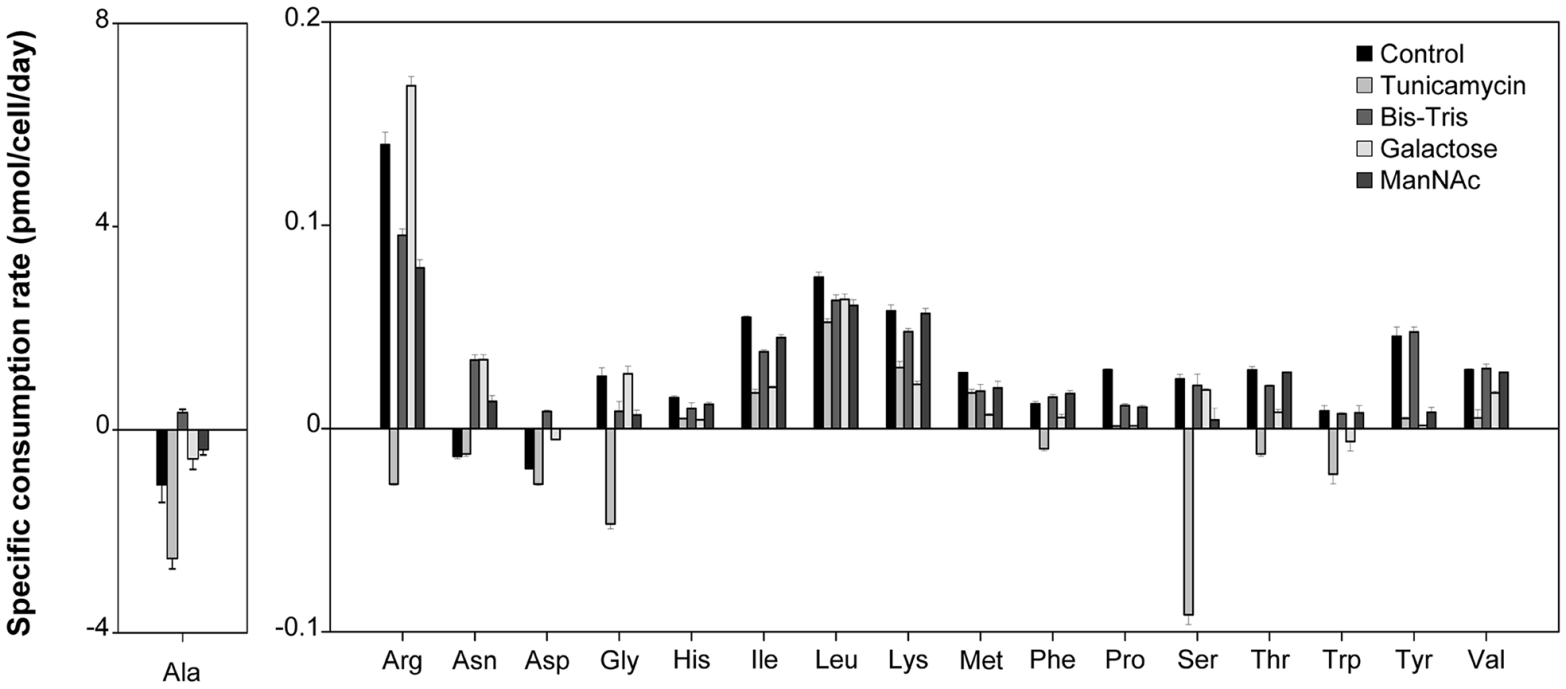
Specific consumption rates of amino acids in serum-free suspended hybridomas supplemented with and without four glycosylation modulators. The specific consumption rates of the amino acids were calculated from 2– 3-day cultures, which were expected before and after the addition of the glycosylation modulators, respectively. Error bars represent the standard deviations calculated from technical triplicate analyses.

Fig. 2 shows the specific consumption rates of individual amino acids for serum-free suspended hybridomas during culture with or without glycosylation modulators from days 2 to 3, as shown in Fig. 1. Tunicamycin treatment resulted in a significant accumulation of alanine, arginine, glycine, serine, and tryptophan, in contrast to the culture with or without the other three glycosylation modulators. It has been reported that the accumulation of serine is attributed to decreased cell growth [22]. In cultures treated with Bis-Tris, galactose, and ManNAc, the consumption of most amino acids showed varying trends during culture without these, except for leucine. This variation in amino acid metabolism is likely the result of changes in diagnostic antibody productivity and glycosylation patterns.

### Effect of Glycosylation Modulators on *N*-Glycosylation of Diagnostic Antibody

To evaluate the effect of glycosylation modulators on the *N*-glycan of diagnostic monoclonal antibody from a hybridomas culture, the antibody produced on day 3 was purified by protein G chromatography (Fig. 1). Cell culture supernatants with viability greater than 80% were harvested to avoid the possibility of impaired glycosylation from sialidase released following cell membrane disruption. The major *N*-glycans released from the diagnostic antibody following PNGase F incubation were analyzed by hydrophilic interaction HPLC column and classified into six groups (G0, G0F, Man, G1F, G2F, G2S1F, and G2S2F) based on their structure.

**Fig. 3:**
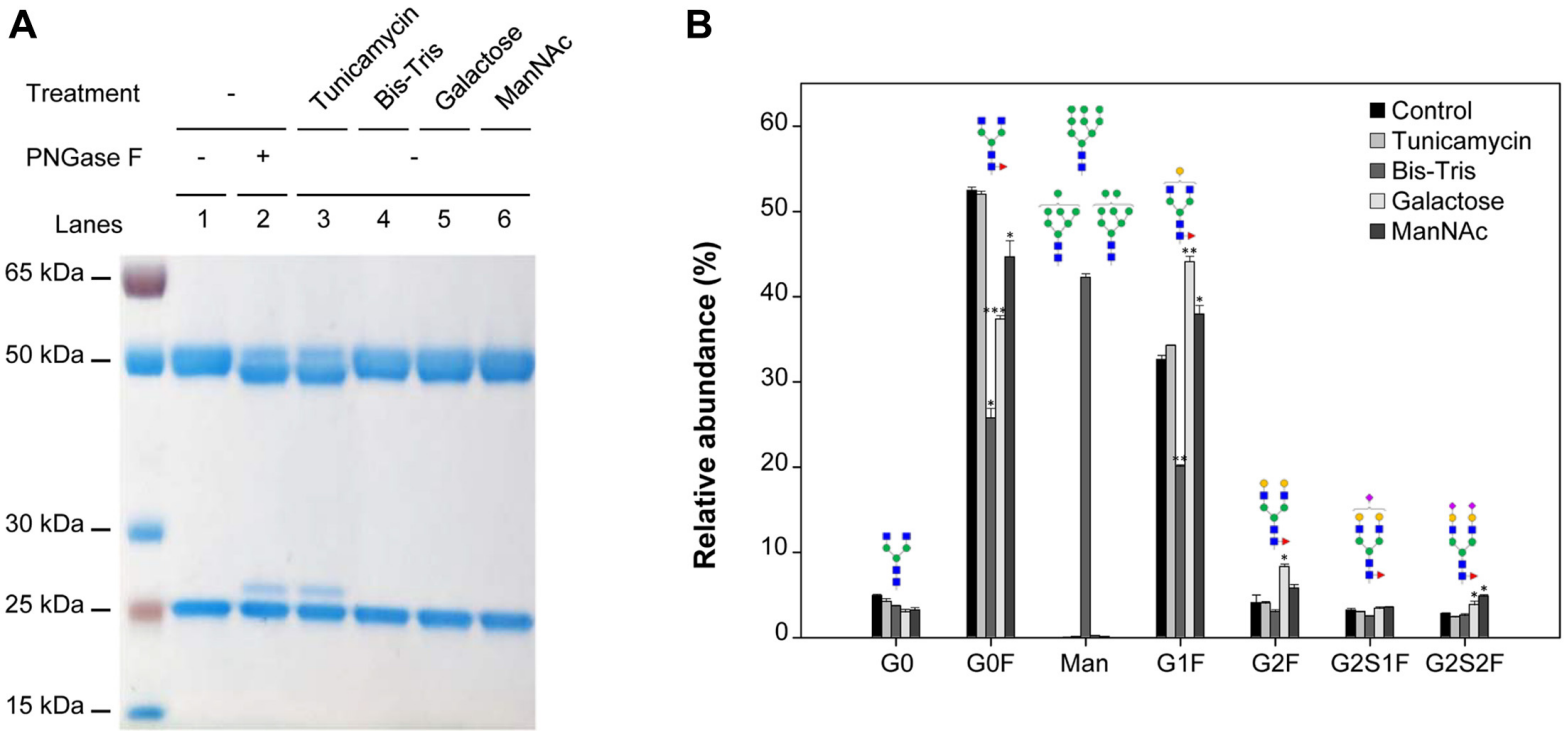
(A) SDS-PAGE analysis of the purified diagnostic antibody produced from hybridomas supplemented with and without four glycosylation modulators, followed by treatment with (+) and without (‒) PNGase F. The diagnostic antibody was purified by protein G affinity chromatography from the culture supernatants harvested on day 3 with cell viability greater than 80% and treated with PNGase F for the release of *N*-glycans. Equal amounts of proteins were loaded under reducing conditions and the gel was stained with Coomassie brilliant blue. (**B**) Hydrophilic interaction HPLC analysis of the major *N*-glycans obtained from the purified diagnostic antibody from hybridomas on day 3. The relative abundance of six major *N*-glycans (G0, G0F, Man, G1F, G2F, G2S1F, and G2S2F) was obtained from cultures with and without four glycosylation modulators. Error bars represent standard deviations calculated from triplicate experiments. *p ≤ 0.05, **p ≤ 0.01, ***p ≤ 0.001.

Fig. 3 shows the expression pattern of the diagnostic antibody as determined by SDS-PAGE analysis and the proportion of major *N*-glycans, which were calculated by integrating the area of each group following UHPLC analysis. The diagnostic antibody produced from the culture with tunicamycin showed a significant increase in the aglycosylated form compared with that treated with or without other glycosylation modulators (Fig. 3A); however, the *N*-glycan profile was similar to that of the control culture (Fig. 3B). Bis-Tris treatment significantly increased the proportion of highly mannosylated *N*-glycans to 42.27% ± 0.5% compared with the control cultures (<0.5%). The sum of the proportions of biantennary galactosylation (G2F, G2S1F, and G2S2F) was 16.22% ± 0.82% following galactose supplementation, which was the highest and higher than that of the control culture (10.04% ± 0.81%) (*p* ≤ 0.05). Compared with the control culture (2.79% ± 0.2%), the sum of the proportions of biantennary sialylation (G2S1F, and G2S2F) significantly increased (4.85% ± 0.58%) following the addition of ManNAc (*p* ≤ 0.05). Taken together, supplementation with the four glycosylation modulators inhibited glycosylation and increased mannosylation, galactosylation, and sialylation in the diagnostic antibody-producing hybridomas.

### Effect of Glycosylation Modulators on Intracellular Nucleotide Sugar Pools

The amount of intracellular nucleotide sugar pools in mammalian cells is important for the *N*-glycosylation process and its availability greatly affects the glycan complexity and final glycosylation profile [23]. To determine whether the addition of glycosylation modulators affected the concentration of intracellular nucleotide sugar in the hybridomas, we quantified the amount of six intracellular nucleotide sugar pools using ion-pair reversed-phase HPLC.

**Fig. 4:**
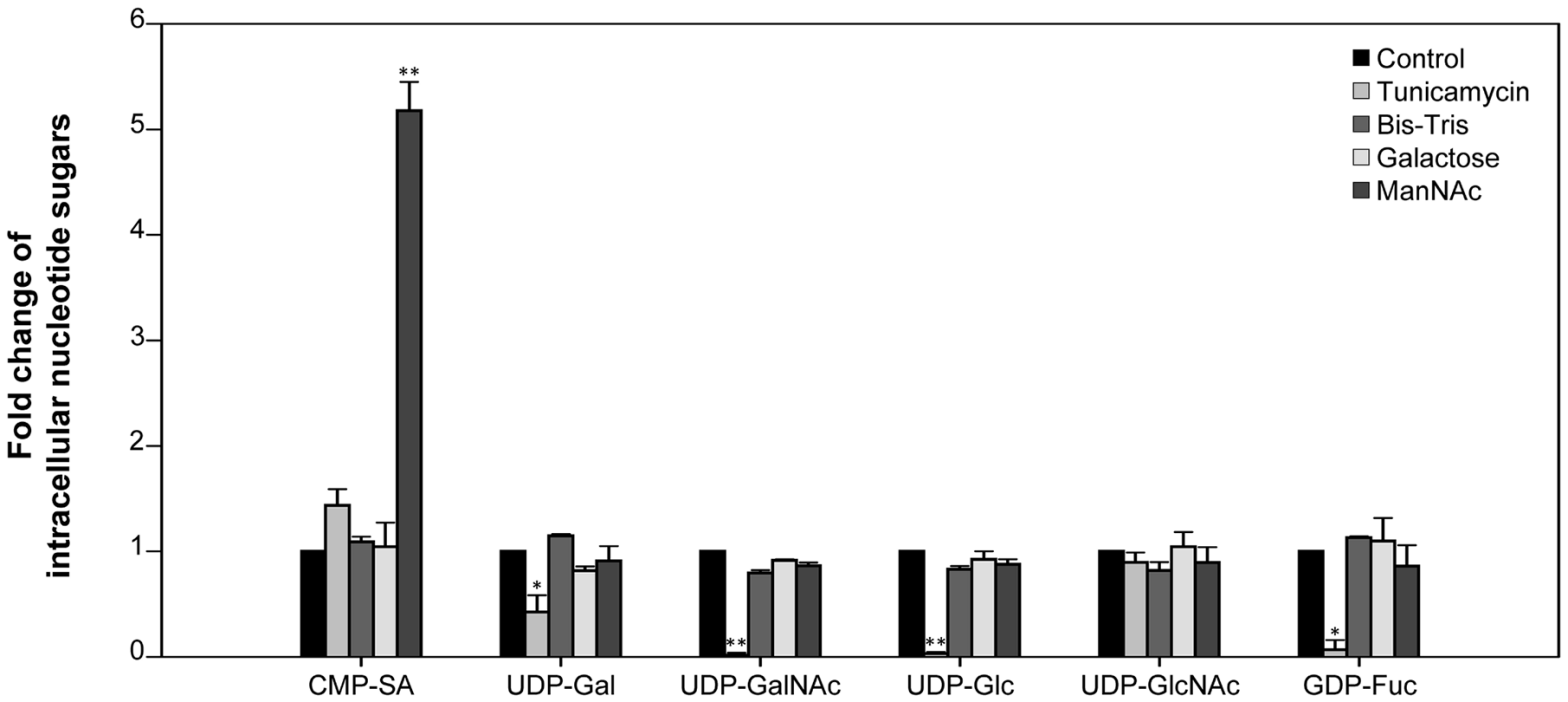
Fold change in intracellular nucleotide sugars in serum-free suspended hybridomas supplemented with and without four glycosylation modulators. Nucleotide sugars were extracted and quantified from cells on day 3 using ion-pair reversed-phase HPLC. The fold change for each nucleotide sugar was calculated based on each nucleotide sugar in the culture without glycosylation modulators. Error bars represent the standard deviations calculated from technical triplicate experiments. *p ≤ 0.05, **p ≤ 0.01

Fig. 4 shows the relative amounts of the six intracellular nucleotide sugars (CMP-SA, UDP-Gal, UDP-GalNAc, UDP-Glc, UDP-GlcNAc, and GDP-Fuc) for the hybridomas in the presence or absence of the four glycosylation modulators on day 3. The fold-change values were calculated based on the number of nucleotide sugars measured in the cell lysates on day 3 of the control culture. Consistent with a previous study examining the effect of ManNAc in rCHO cells [20], ManNAc addition significantly increased the concentration of CMP-SA, a nucleotide sugar directly used for sialylation in hybridomas compared with the control culture. Notably, a significant decrease in UDP-Gal, UDP-GalNAc, UDP-Glc, and GDP-Fuc was observed in the tunicamycin-treated culture, which may have been related to aglycosylated antibody production. Treatment with Bis-Tris increased the proportion of highly mannosylated *N*-glycans, whereas there was no significant change in the concentration of intracellular nucleotide sugars (*p* > 0.05).

### Binding Affinity of the Diagnostic Antibody with Various *N*-Glycosylation Patterns

To determine whether the diagnostic antibody with various major *N*-glycan profiles affects the binding affinity with the target antigen, we performed an ELISA with the purified diagnostic antibody and positive quality control materials produced by the mouse ascites method.

**Fig. 5:**
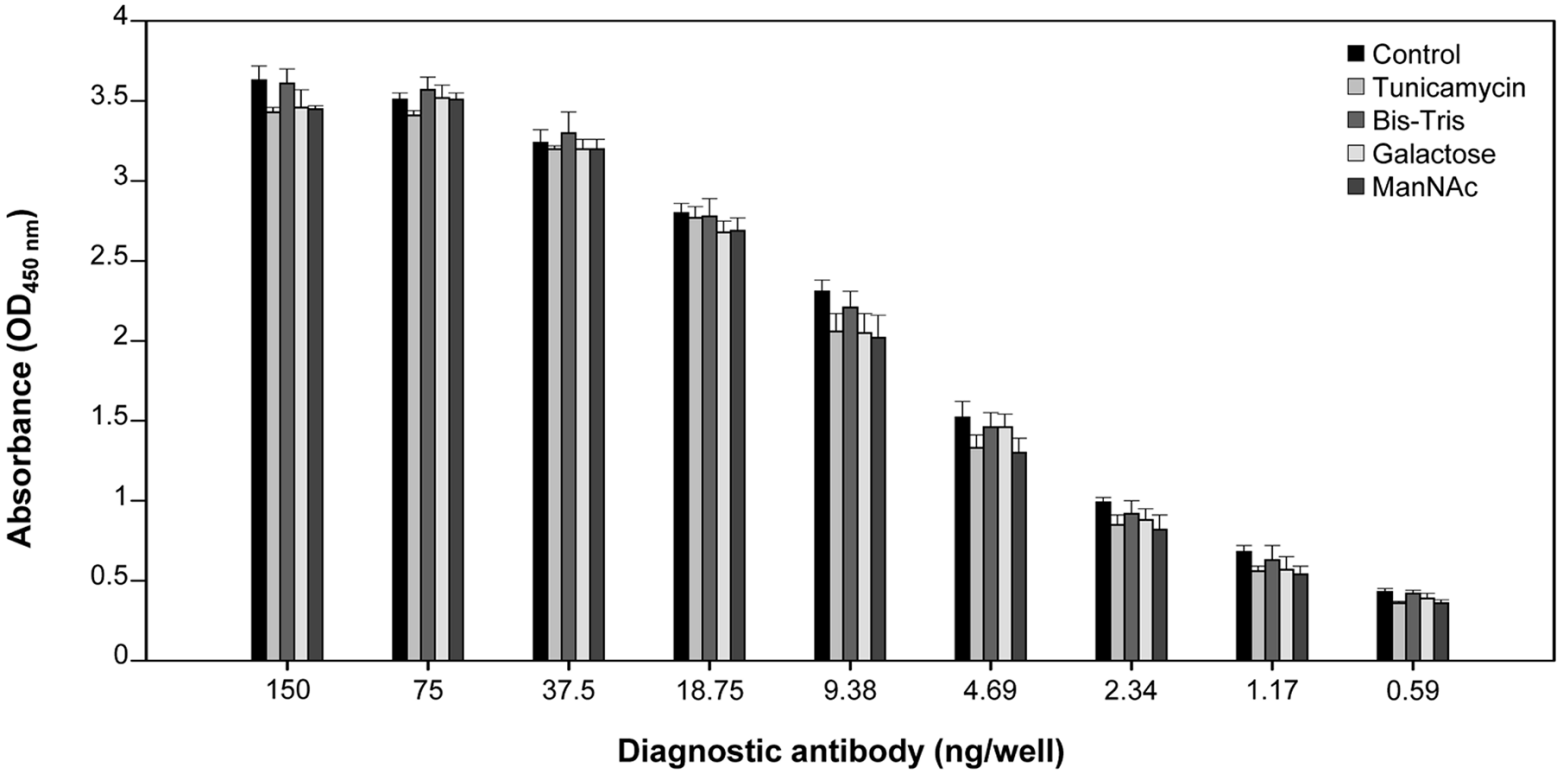
The binding affinity assay for the purified diagnostic antibody with a specific target antigen. The absorbance (OD_450nm_) was measured using a conventional ELISA method with a diagnostic antibody produced from hybridomas in the absence and presence of four glycosylation modulators. Error bars represent standard deviations calculated from data obtained in technical triplicate experiments.

Fig. 5 shows the results of the binding affinity assay in the presence of different diagnostic antibody concentrations produced from cultures with and without the four other glycosylation modulators. The pattern of the binding affinity assay with the diagnostic antibody produced from serum-free suspended hybridomas attempted was similar to that from mouse ascites, which was used as a positive control (data not shown). Regardless of the type of *N*-glycan profile, the binding affinity between the diagnostic antibody and its target antigen was highly dose-dependent. Unexpectedly, the type of *N*-glycan profile did not have a significant effect on binding to the target antigen and even the aglycosylated antibody exhibited a similar binding affinity. Taken together, the results indicate that the modulation of the *N*-glycan profile of the antibody by the altered culture environment in serum-free suspension hybridomas does not affect its function in a diagnostic assay.

The use of serum-free suspension culture for hybridomas in the production of diagnostic antibody has the benefits of high productivity and the elimination of contamination from nonspecific antibodies that are present in serum. Generally, it is very difficult to maintain the glycosylation of an antibody produced in serum-free suspension cultures of mammalian cells, as it is dependent on the culture environment and prone to cell death. We demonstrated that the *N*-glycan profile of the diagnostic antibody produced by serum-free suspended hybridomas is not an important factor in binding affinity, which is a key factor for diagnostic assay (Fig. 4). These results will enable us to utilize culture conditions that previously had negative effects on *N*-glycans in rCHO cell culture, for antibody production. For example, sodium butyrate is a well-known additive that enhances *q*_p_, but it is not widely used in the production of therapeutic proteins in rCHO cell culture because of its negative impact on *N*-glycosylation [24, 25]. Because there is no relationship between *N*-glycosylation and the activity of the diagnostic antibody, we can focus on maximizing its production using various additives and culture processes that are not used for rCHO cell culture.

Glycosylation of the Fc region in the therapeutic monoclonal antibody significantly affects the Fc-mediated effector functions of the antibody, such as antibody-dependent and complement-dependent cytotoxicity [26]. Therefore, precise control of glycosylation in therapeutic monoclonal antibody is necessary to maintain consistent function and effectiveness. In contrast, the diagnostic monoclonal antibody should be optimized for antigen binding affinity and specificity of the Fab region rather than the immune effect of the Fc region. Although it can occur in the Fab and Fc regions, glycosylation of the monoclonal antibody predominantly exists in the Fc region [27]. Therefore, glycosylation is not likely to significantly affect the diagnostic use of the monoclonal antibody, which is consistent with our findings. These results suggest that the importance of glycosylation of the Fc region in the monoclonal antibody may vary depending on the purpose for which the antibody is used.

In this study, the introduction of serum-free suspension culture resulted in a 5.7-fold increase in diagnostic antibody production in hybridomas (Fig. 1); however, the volumetric titer of the diagnostic antibody produced from the hybridomas tends to be relatively low, less than 1 g/L, compared with the high titers achieved (10 g/L) for recombinant therapeutic antibody in rCHO cells using high expression vectors. Recent studies successfully sequenced the nucleotides of antibody from hybridomas and produced them in the form of recombinant antibody in mammalian cells [28-30]. The recombinant antibody produced in mammalian cells is expected to differ in *N*-glycan composition from the antibody produced in hybridomas in our study; however, it is expected to have similar biological function when used as a diagnostic antibody. Based on our finding, establishing recombinant diagnostic antibody-producing mammalian cells with the same antibody binding affinity as hybridomas is an effective method to achieve high titers of diagnostic antibody.

In conclusion, we produced a diagnostic antibody with various *N*-glycan profiles from a serum-free suspension hybridomas culture with glycosylation modulators. We also demonstrated that *N*-glycosylation of diagnostic antibody did not significantly affect binding affinity, which is useful for the efficient production and quality control of diagnostic antibody.
